# Le Fevre’s Diagnosis

**Published:** 1906-01

**Authors:** 


LE FEVRE'S DIAGNOSIS. A Manual on Physical Diagnosis, including Diseases of the Thoracic and Abdominal Organs. For Students and Physicians. By Egbert LeFevre, M.D., Professor of Clinical Medicine and Therapeutics in the University and Bellevue Hospital Medical College, Attending Physician to Bellevue Hospital and to St. Luke's Hospital, New York. New (2d) edition, thoroughly revised and much enlarged. In one 12mo volume of 479 pages with 102 engravings and 6 full page plates in black and colors. Cloth, $2.25, net. Lea Brothers & Co., Publishers, Philadelphia and New York.

This new edition has been thoroughly revised, and some sections have been entirely rewritten, the general plan of the book however has been preserved. It was designed for the student and practitioner and represents the methods developed in the large experience of Dr. Le Fevre as a teacher.

The scope of the work has been kept to its title, including the subjects of Inspection, Palpation, Percussion and Auscultation.

